# High-intensity focused ultrasound combined with syndrome differentiation-based individualized herbal therapy and 12-month outcomes in patients with adenomyosis: a prospective non-randomized study

**DOI:** 10.3389/fmed.2026.1758003

**Published:** 2026-08-24

**Authors:** Cuiling Zhang, Lijun Zhang, Shuping Zhao, Yanyan Huang, Liangqian Zhao, Rongxing Wang, Yuling Wang, Deyan Sun, Yongsheng Ma, Qiuling Shi

**Affiliations:** 1 State Key Laboratory of Ultrasound in Medicine and Engineering, College of Biomedical Engineering, Chongqing Medical University, Chongqing, China; 2 Department of Gynecology, Linyi Hospital of Traditional Chinese Medicine, Linyi, Shandong, China; 3 Department of Gynecology, Qingdao Women and Children's Hospital, Qingdao, Shandong, China; 4 Department of Neurosurgery and Glioma Medical Research Center, Southwest Hospital, Third Military Medical University (Army Medical University), Chongqing, China; 5 Department of Pharmacy, Linyi Hospital of Traditional Chinese Medicine, Linyi, Shandong, China; 6 Department of Traditional Chinese Medicine, Linyi Hospital of Traditional Chinese Medicine, Linyi, Shandong, China

**Keywords:** adenomyosis, dysmenorrhoea, high intensity focused ultrasound, TCM, traditional Chinese medicine

## Abstract

**Objective:**

To evaluate the association of high-intensity focused ultrasound (HIFU) combined with syndrome differentiation-based individualized traditional Chinese medicine (TCM) herbal therapy with 12-month clinical outcomes compared with HIFU monotherapy in patients with adenomyosis (AM).

**Methods:**

This prospective non-randomized, patient-preference, parallel-controlled study enrolled 88 patients with adenomyosis between November 2021 and November 2023; 87 completed 12-month follow-up and were analyzed (HIFU, *n* = 45; HIFU+TCM, *n* = 42). TCM prescriptions tailored for damp-heat and blood stasis obstruction, qi stagnation, or cold coagulation syndromes were administered for 3 months after HIFU. The primary outcome was the 12-month dysmenorrhoea numerical rating scale (NRS) score. Secondary outcomes were uterine volume, lesion volume, serum CA125, adverse events, and symptom recurrence. The primary analysis used multivariable-adjusted linear mixed-effects models. Stabilized inverse probability of treatment weighting (IPTW), with 1st/99th percentile trimming, was used as a sensitivity analysis; baseline balance was assessed using standardized mean differences.

**Results:**

At 1 month, the adjusted between-group NRS difference was not significant (*P* = 0.174). From 3 to 12 months, the HIFU+TCM group had lower adjusted NRS scores than the HIFU group (all *P* < 0.05). Uterine volume was smaller at 6, 9, and 12 months, and lesion volume at 9 and 12 months. IPTW sensitivity analyses supported these findings. Serum CA125 did not differ significantly between groups at any time point (12 months, *P* = 0.362). Adverse events were mild and comparable. Symptom recurrence occurred in 4/45 (8.89%) patients receiving HIFU and 6/42 (14.29%) receiving HIFU+TCM (*P* = 0.492).

**Conclusion:**

In this prospective non-randomized study, HIFU combined with syndrome differentiation-based individualized TCM herbal therapy was associated with greater improvement in dysmenorrhoea and greater reductions in uterine and lesion volumes over 12 months than HIFU alone, but not with lower symptom recurrence. These preliminary associations require confirmation in adequately powered randomized controlled trials.

## Introduction

Adenomyosis (AM) is characterized by ectopic endometrial invasion into the myometrium and imposes a substantial burden on quality of life through progressive dysmenorrhoea and menorrhagia (1). Although hysterectomy remains the only curative treatment (2), it is unsuitable for patients who wish to preserve the uterus. Uterus-preserving strategies remain challenging in clinical practice. Pharmacological treatments (such as GnRHa, dienogest and LNG-IUD) can control symptoms, but their clinical utility is limited by short-term efficacy, adverse effects, and recurrence after treatment discontinuation, especially in women requiring long-term management (3–6). Adenomyomectomy, although uterus-preserving, is invasive and associated with high recurrence rates.

High-intensity focused ultrasound (HIFU), a non-invasive treatment, has shown favorable safety and symptom-control efficacy in patients with adenomyosis by ablating lesions through thermal effects (4, 5). However, its clinical application is constrained by several limitations. The ill-defined boundaries of adenomyotic lesions often result in incomplete ablation, leading to residual symptoms and subsequent recurrence (6–9). Long-term follow-up studies have indicated that symptom recurrence may occur within 2–3 years after HIFU alone, highlighting the need to explore adjunctive therapies that may support longer-term disease control (5, 7, 9).

Traditional Chinese medicine (TCM) has a long history in the treatment of AM, with blood stasis regarded as a core pathological mechanism and therapeutic principles focused on promoting blood circulation, resolving stasis, and relieving pain (10, 11). Contemporary studies in adenomyosis and related dysmenorrhoeic disorders suggest that herbal therapy may alleviate pain symptoms and support reduction of lesion burden, although heterogeneity in formulation and study quality remains substantial (12–14). In addition, pharmacological investigations have shown that components such as Radix Paeoniae Rubra and Panax notoginseng may exert anti-inflammatory, immunomodulatory, and antiproliferative effects relevant to ectopic endometrial activity (15–18). However, existing TCM research in AM has primarily relied on fixed prescriptions, neglecting individualized syndrome differentiation, which is a cornerstone of TCM that tailors treatment according to a patient's specific pathological condition (10–14).

Notably, the potential complementarity between HIFU and TCM remains insufficiently explored. For AM specifically, available studies of HIFU combined with TCM have mainly used standardized formulas. One study using Wenjing Tongzhu Formula after HIFU reported improved short-term dysmenorrhoea relief but no significant long-term difference in lesion regression (13). Another study using Xuefu Zhuyu Decoction reported improvement in 6-month CA125 reduction but did not analyse syndrome-specific efficacy (14). A recent systematic review and meta-analysis also suggested that herbal medicine may improve short- and medium-term post-HIFU outcomes in AM, whereas evidence for recurrence prevention remains insufficient (15).

HIFU directly ablates macroscopic lesions, whereas TCM may theoretically target residual microscopic ectopic tissue, regulate the local uterine microenvironment, and enhance immune homeostasis, thereby potentially complementing the effects of HIFU (10, 15–19). However, current studies of HIFU combined with TCM still largely rely on standardized formulas (13, 14, 20), failing to incorporate the core TCM principle of syndrome differentiation to optimize individual therapeutic responses. Furthermore, few longitudinal studies with at least 12 months of follow-up have systematically evaluated symptom relief, lesion regression, and recurrence after HIFU combined with syndrome differentiation-based TCM.

This study aimed to investigate whether HIFU combined with syndrome differentiation-based TCM was associated with differences in dysmenorrhoea, uterine volume, lesion volume, serum CA125, and 12-month symptom recurrence compared with HIFU alone in patients with AM.

## Materials and methods

### Study design

This prospective, non-randomized, patient-preference parallel-controlled study was registered with the Chinese Clinical Trial Registry and approved by the ethics committee of Linyi Hospital of Traditional Chinese Medicine of Shandong (No. LYSZYYY20220056). The study was conducted in accordance with the Declaration of Helsinki. All patients were fully informed of the study design, including the non-randomized grouping method (group allocation based on patient preference), potential risks, benefits and follow-up requirements, and they all provided written informed consent using a standardized consent form.

Based on preliminary clinical data regarding changes in 12-month dysmenorrhoea NRS score (the primary outcome), we calculated the required sample size using G^*^Power 3.1. We set α = 0.05, statistical power = 80%, and assumed an intergroup mean difference of 1.4 points in NRS score with a standard deviation of 1.2 points. The calculated minimum sample size for each group was 40 participants. Considering an expected 10% participant dropout rate during 12-month follow-up, we planned to enroll 44 patients in each group (88 participants in total).

### Participants

The diagnostic criterion for AM was confirmation by US and magnetic resonance imaging (MRI) based on the following features: (1) diffuse or focal thickening of the myometrium with ill-defined boundaries, (2) heterogeneous echogenicity on US or high signal intensity on T2-weighted MRI and (3) presence of ectopic endometrial glands and stroma within the myometrium on histopathology, when available (1, 3, 20).

The inclusion criteria were as follows: (1) progressive dysmenorrhoea and/or menorrhagia without dyspareunia, (2) diagnosis of AM confirmed by US and MRI, (3) presence of a safe acoustic window and (4) myometrial thickness ≥3 cm (4, 7).

The exclusion criteria were as follows: (1) pregnancy, lactation or menstruation; (2) acute pelvic inflammation; (3) suspected gynecological malignancy; (4) history of radiotherapy; (5) inability to communicate with physicians during the procedure; and (6) presence of dyspareunia (to reduce heterogeneity in subjective pain outcomes).

The criteria for termination of study participation were as follows: (1) occurrence of severe adverse events during treatment or follow-up (e.g., grade 3 or higher pain unrelieved by medication, severe allergic reactions to TCM herbs, HIFU-induced skin burns requiring surgical intervention or treatment-related acute organ dysfunction); (2) voluntary withdrawal of informed consent by the participant because of personal reasons (e.g., inability to continue follow-up or dissatisfaction with treatment progress); (3) loss to follow-up for more than 2 consecutive months, defined as failure to complete scheduled assessments at 3, 6, 9 or 12 months without valid reasons and inability to establish contact; (4) violation of the study protocol by the participant (e.g., non-compliance with TCM medication for more than 14 consecutive days or receipt of other treatments for AM [such as GnRHa injection or adenomyomectomy] during the study period); and (5) newly diagnosed gynecological malignancy or other serious diseases (e.g., severe cardiovascular disease or malignant tumor) during follow-up that could affect evaluation of study outcomes or compromise participant safety.

### High-intensity focused ultrasound procedure

The device used for HIFU treatment was the Focused Ultrasound Tumor Therapeutic System (Model JC200; Chongqing Haifu Medical Technology Co., Ltd.). The HIFU treatment protocol followed established ultrasound-guided HIFU practice for gynecological diseases (20, 21). Patients underwent bowel preparation, skin preparation and bladder catheterisation before treatment. High-intensity focused US was performed under conscious sedation with the patient in the prone position.

Power adjustment was performed as follows: the initial power setting was 300–350 W and was adjusted according to real-time grayscale changes observed on monitoring US. (1) If grayscale increased by < 20% within 30 s, the power was increased by 50 W (maximum: 400 W). (2) If excessive pain or abnormal tissue echogenicity occurred, the power was reduced by 50 W.

Termination criteria for the procedure were as follows: (1) grayscale change within the lesion reached ≥80% on US, (2) no blood supply was detected on contrast-enhanced US or (3) the patient experienced intolerable pain despite power reduction (20, 21).

Treatment parameters were as follows: mean treatment duration of 112 ± 24 min, total energy of 1,280 ± 320 kJ and non-perfused volume ratio of 78% ± 11%. All procedures were performed by operators with more than 5 years of experience.

### Administration of traditional Chinese medicine

Traditional Chinese medicine treatment was initiated 1 day after HIFU and continued for 3 months. Patients were classified into TCM syndromes according to symptoms, tongue characteristics and pulse findings.

Patients with damp-heat and blood stasis obstruction syndrome, characterized by a dark purple tongue with petechiae and a choppy pulse, were treated with Radix Paeoniae Rubra 15 g, Panax notoginseng 10 g, Cortex Moutan 12 g, Rhizoma Corydalis 15 g and Herba Taraxaci 20 g, decocted daily.

Patients with qi stagnation syndrome, characterized by distending pain and a wiry pulse, were treated with modified Chaihu Shugan powder consisting of Radix Bupleuri 12 g, Rhizoma Cyperi 10 g, Fructus Aurantii 10 g and Radix Angelicae Sinensis 12 g, decocted daily.

Patients with cold coagulation syndrome, characterized by cold pain relieved by heat and a white, greasy tongue coating, were treated with modified Wenjing decoction consisting of Ramulus Cinnamomi 10 g, Poria cocos 15 g, Rhizoma Zingiberis 6 g and Radix Angelicae Sinensis 12 g, decocted daily.

Prescriptions were adjusted monthly according to changes in syndrome classification. All interventions were oral herbal decoctions. Medication adherence was monitored via two combined methods: (1) monthly weighing of residual herbal materials from each patient's decoction to calculate actual dosage intake; (2) standardized patient medication diaries to record daily medication completion. The overall medication adherence rate among all included patients exceeded 90% (10, 12).

### Treatment evaluation

The primary outcome was dysmenorrhoea severity measured using the NRS at 12 months after HIFU treatment. Secondary outcomes included uterine volume, adenomyotic lesion volume and serum CA125 levels.

Numerical rating scale scores were used to assess dysmenorrhoea severity. Pain intensity was scored on a scale of 0–10 and categorized as follows: no pain (0 points), mild pain (1–3 points), moderate pain affecting sleep (4–6 points) and severe pain that was difficult to tolerate and affected appetite and sleep (7–10 points) (22). The clinical relevance of the NRS lies in its ability to quantitatively assess dysmenorrhoea severity. Patients with AM typically present with progressive dysmenorrhoea, and changes in NRS scores directly reflect the degree of symptom relief after treatment, making this a key indicator of improvement in quality of life. Numerical rating scale scores were assessed at baseline (1 week before HIFU intervention) and at 1, 3, 6, 9 and 12 months after HIFU intervention.

Uterine and lesion volumes were assessed using US (Model EPIQ 7, Philips) and MRI (3.0 T, Signa HDxt, GE Healthcare) at each follow-up visit. Uterine volume and lesion volume are important anatomical indicators in AM. Uterine volume reflects the overall extent of myometrial involvement by ectopic endometrial tissue, whereas lesion volume directly reflects the size of localized adenomyotic lesions. Reductions in these volumes indicate regression of the disease and treatment efficacy.

For US assessment, uterine and lesion volumes were calculated using the ellipsoid formula: 0.5233 × length × anteroposterior diameter × transverse diameter (unit: cm^3^).

For MRI assessment, lesion boundaries were manually traced on T2-weighted images, and lesion volume was calculated using ImageJ (unit: cm^3^). In this study, ultrasound-derived volume data served as the primary dataset for all statistical analyses, while MRI measurements were exclusively applied for cross-validation and reliability verification. Unified measurement standards and operating protocols were implemented for all imaging examinations throughout follow-up.

All measurements were performed by three trained physicians, with an intraclass correlation coefficient >0.85 (Table 1). Both uterine volume and lesion volume were measured at baseline (1 week before HIFU intervention) and at 1, 3, 6, 9 and 12 months after HIFU intervention. Representative US and MRI images of lesions are provided in Supplementary Figure 1.

**Table 1:** Consistency validation between MRI and ultrasound measurements using Bland–Altman analysis.

Measurement	Intraclass correlation coefficient (ICC)	Bland–Altman analysis results
Uterine volume (cm^3^)	0.92	Mean difference between MRI and ultrasound: 5.2 cm^3^ (95% CI: −12.8 to 23.2 cm^3^); 95% limits of agreement: −31.6 to 42.0 cm^3^
Lesion volume (cm^3^)	0.89	Mean difference between MRI and ultrasound: 3.8 cm^3^ (95% CI: −9.6 to 17.2 cm^3^); 95% limits of agreement: −26.4 to 34.0 cm^3^

High agreement between MRI and ultrasound measurements is confirmed by high ICC values (>0.85) and narrow 95% limits of agreement, indicating consistent volume assessment between the two methods.

Recurrence was defined as either a return of the NRS score to ≥4 points (moderate pain) or an increase of ≥50% from the nadir value after initial improvement.

Serum CA125 levels were measured using a chemiluminescent immunoassay kit (Model Architect CA125 II, Abbott Laboratories) on an automated analyser (Model Architect i2000SR). The detection range was 0–500 U/mL, with intra-assay and inter-assay coefficients of variation < 5%. Cancer antigen 125 is a serological marker associated with AM. Although not specific to AM, CA125 levels are elevated in many patients with the disease, and reductions in CA125 after treatment may indirectly reflect decreased ectopic endometrial activity and disease severity (23–25). Serum CA125 levels were measured at baseline (1 week before HIFU intervention) and at 1, 3, 6, 9 and 12 months after HIFU intervention.

### Statistical analysis

Continuous variables are presented as mean ± standard deviation or median (interquartile range), as appropriate. A linear mixed-effects model with random intercept was constructed for longitudinal data analysis to account for within-participant repeated-measurement correlation and incomplete follow-up data under the missing at random assumption. Fixed factors included treatment group, follow-up time, and group-time interaction. The random factor was individual participant ID. Adjusted mean differences (AMD) and 95% confidence intervals (CIs) were reported together with *P* values. Multivariable adjustment was performed for age, disease duration, baseline NRS score, baseline uterine volume, baseline lesion volume, and adenomyosis phenotype.

Inverse probability of treatment weighting (IPTW) based on the propensity score was applied as a sensitivity analysis. To reduce the risk of overfitting and unstable weights in this modest-sized cohort, the propensity score model was prespecified to include clinically relevant baseline variables associated with treatment selection or outcome: age, disease duration, baseline NRS score, baseline uterine volume, baseline lesion volume, baseline CA125, hypermenorrhea, adenomyosis phenotype, coexisting endometriosis, coexisting uterine fibroids, prior cesarean section, and TCM syndrome type. Propensity scores were estimated using logistic regression. Stabilized average treatment effect weights were used, and weights were trimmed at the 1st and 99th percentiles. Before weighting, the largest absolute SMD was 0.18; after weighting, all absolute SMDs were < 0.10, with a maximum of 0.07. The stabilized weights had a median of 0.98 (interquartile range: 0.91–1.06), range 0.63–1.54 after trimming, and no extreme-weight concentration was observed. The effective sample size after weighting was 43.6 in the HIFU group and 39.8 in the HIFU+TCM group. Baseline balance was assessed primarily using SMDs rather than statistical significance testing.

Categorical variables were analyzed using the χ^2^ test. A *P*-value < 0.05 was considered statistically significant. Statistical analyses were performed using SPSS 26.0 and R 4.2.1. Normality and sphericity assumptions were assessed before modeling, and non-normally distributed variables (CA125) were log-transformed before analysis.

## Results

### Patient characteristics

**Figure 1:**
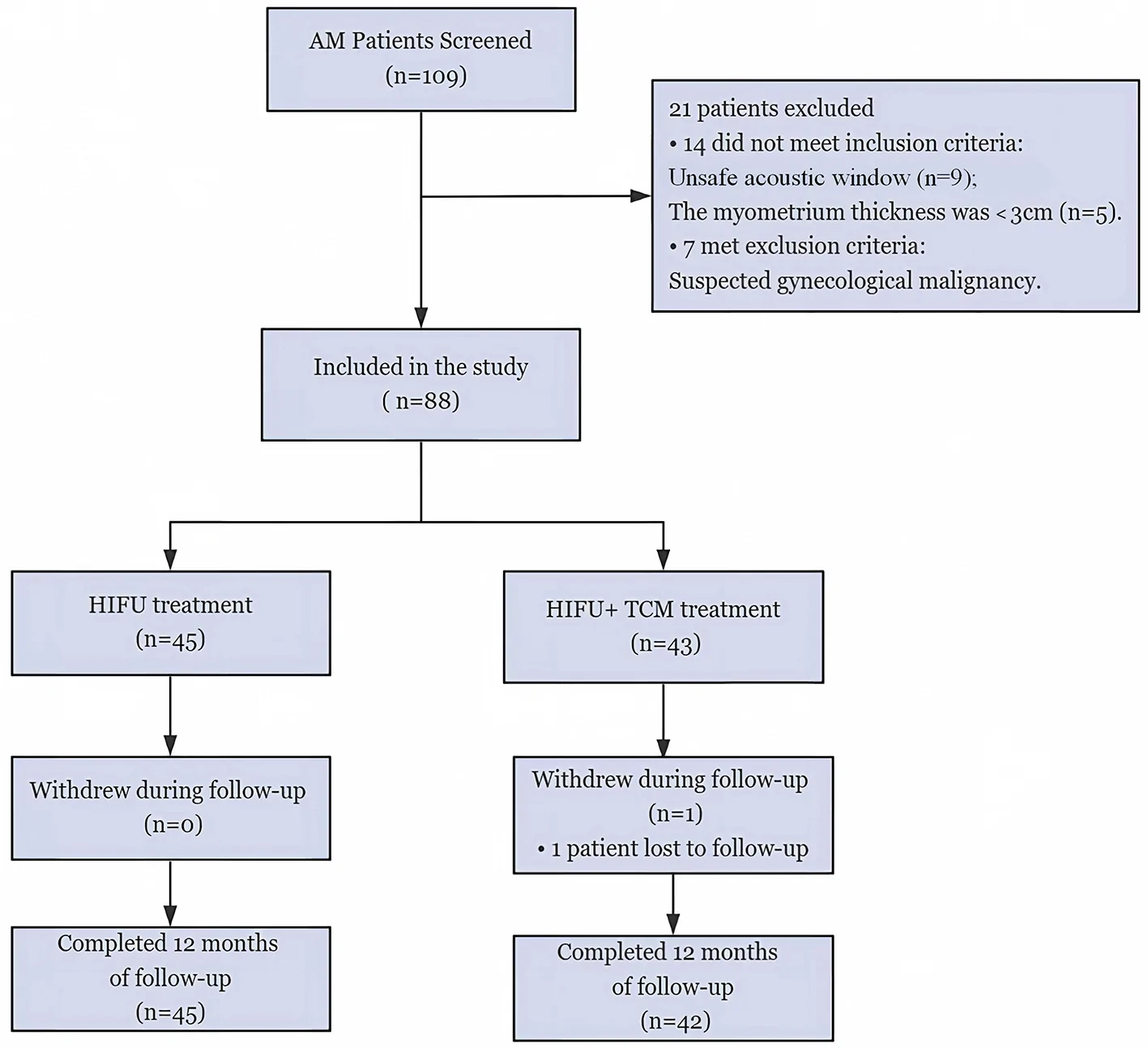
Study flow chart. A total of 109 women with suspected adenomyosis were screened. 21 were excluded before enrolment. 88 eligible patients were enrolled and allocated according to patient preference to the HIFU (High-Intensity Focused Ultrasound) group (*n* = 45) or HIFU + TCM (Traditional Chinese Medicine) group (*n* = 43). One patient in the HIFU + TCM group was lost to follow-up after the 6-month visit. Finally, 87 patients completed the 12-month follow-up and were included in the analysis.

Between November 2021 and November 2023, 109 women with suspected AM were screened, and 88 eligible patients were enrolled, as shown in Figure 1. Of the 109 screened patients, 21 were excluded before enrolment. The non-randomized design was adopted because some patients strongly preferred TCM adjuvant therapy after HIFU, whereas others declined TCM treatment, making randomization impractical in this single-center exploratory study. Patients were therefore divided into two groups according to their preferences: the HIFU group (*n* = 45) and the HIFU + TCM group (*n* = 43).

One patient in the combination group was lost to follow-up after the 6-month visit, resulting in 87 patients completing the 12-month follow-up (HIFU group: 45 patients; HIFU + TCM group: 42 patients). Baseline demographic and disease characteristics, including SMD values before and after IPTW for all propensity-score covariates, are presented in Table 2. Before weighting, the two groups were broadly comparable, with absolute SMDs ≤ 0.18 across baseline variables. After IPTW, all absolute SMDs were < 0.10, supporting satisfactory covariate balance. There were no material imbalances after weighting in age, body size, menstrual characteristics, disease duration, adenomyosis phenotype, baseline symptom severity, uterine volume, lesion volume, or serum CA125.

**Table 2:** Baseline patient demographics and disease characteristics.

Variables	HIFU (*n* = 45)	HIFU + TCM (*n* = 42)	SMD before IPTW	SMD after IPTW	*P* value
Age (years)	42.58 +/− 6.07	43.50 +/− 5.69	0.07	0.03	0.424
Height (cm)	161.53 +/− 4.98	161.21 +/− 4.45	0.06	0.02	0.456
Weight (kg)	63.13 +/− 6.41	63.49 +/− 6.40	0.08	0.04	0.912
Marital status					0.502
Unmarried	2 (4.44%)	1 (2.38%)			–
Married	41 (91.12%)	39 (92.86%)			–
Divorced	2 (4.44%)	2 (4.76%)	0.06	0.03	–
Cesarean section					0.652
Childless	2 (4.44%)	1 (2.38%)			–
No	34 (75.56%)	33 (78.57%)			–
Yes	9 (20.00%)	8 (19.05%)	0.15	0.07	–
Hypermenorrhea			0.15	0.06	0.497
No	12 (26.67%)	14 (33.33%)			–
Yes	33 (73.33%)	28 (66.67%)			–
Comorbidities					–
Hypertension (*n*)	8	7	0.03	0.02	0.892
Diabetes mellitus (*n*)	3	2	0.06	0.04	0.765
Disease duration (years)	4.2 +/− 2.1	3.9 +/− 1.8	0.15	0.06	0.512
Menstrual regularity			0.04	0.02	0.903
Regular (*n*)	38	36			-
Irregular (*n*)	7	6			-
Adenomyosis phenotype			0.04	0.03	0.791
Diffuse type (*n*)	39 (86.7%)	37 (88.1%)			-
Focal type (*n*)	6 (13.3%)	5 (11.9%)			-
Coexisting uterine fibroids	11 (24.4%)	10 (23.8%)	0.02	0.02	0.827
Coexisting endometriosis	7 (15.6%)	6 (14.3%)	0.04	0.03	0.713
Prior hormonal therapy within 3 months	0 (0%)	0 (0%)	0.00	0.00	1.000
TCM syndrome types			0.05	0.04	0.967
Damp-heat and blood stasis obstruction (*n*)	22	20			-
Qi stagnation (*n*)	15	14			-
Cold coagulation (*n*)	8	8			-
NRS score	6.29 +/− 1.53	6.21 +/− 1.30	0.06	0.03	0.718
Uterine volume (cm^3^)	320.38 +/− 239.65	279.87 +/− 113.85	0.18	0.07	0.607
Lesion volume (cm^3^)	104.50 +/− 127.75	90.09 +/− 72.26	0.14	0.06	0.970
CA125 (U/mL)	112.59 +/− 96.51	99.98 +/− 71.51	0.15	0.05	0.721

SMD, standardized mean difference; IPTW, inverse probability of treatment weighting. SMDs are shown for all propensity-score covariates; for multi-level categorical variables, the SMD represents the overall variable.

### Comparison of dysmenorrhoea between the two groups

**Figure 2:**
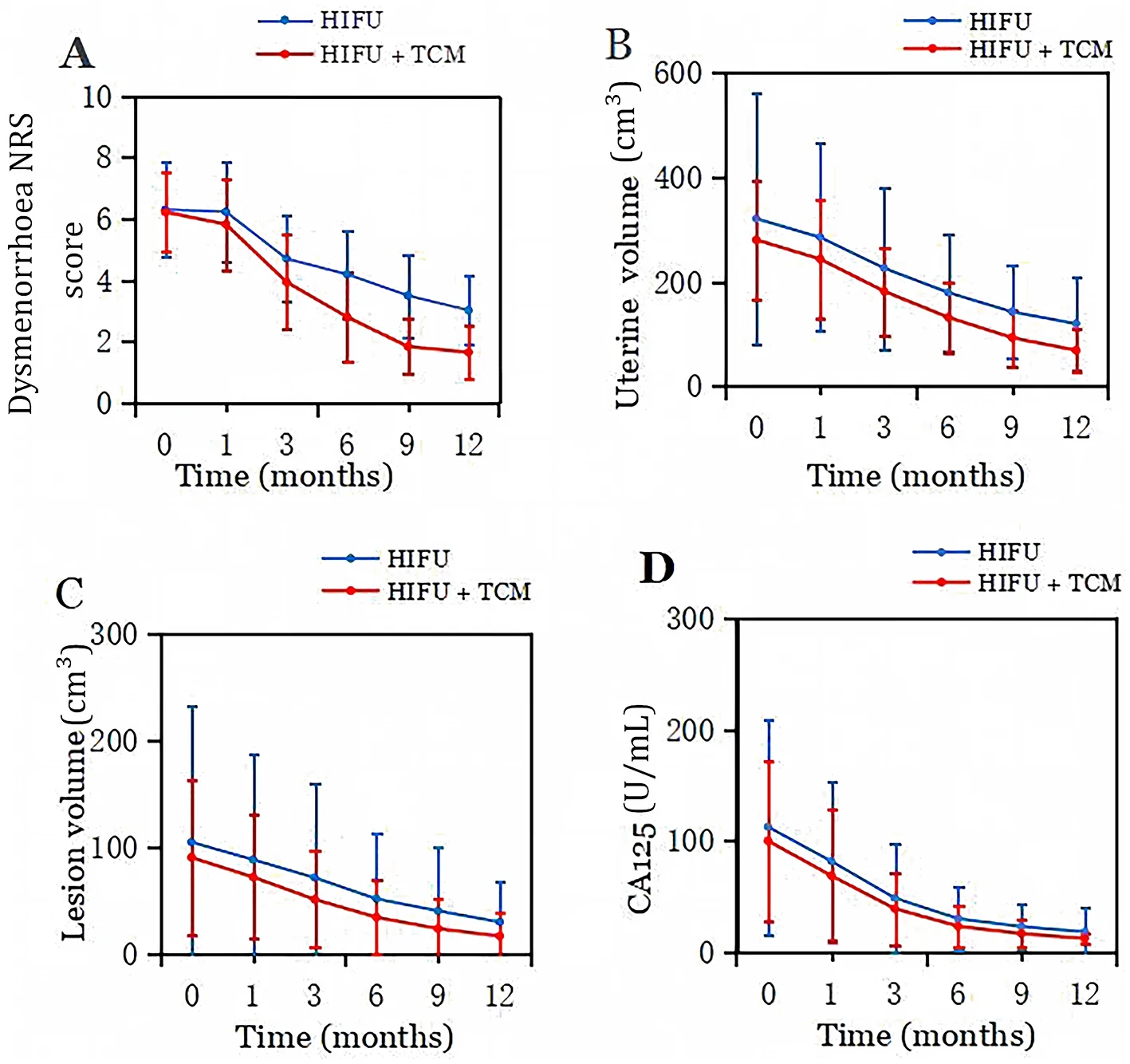
Longitudinal changes in **(A)** dysmenorrhoea NRS (Numerical Rating Scale) score, **(B)** uterine volume (cm^3^), **(C)** adenomyotic lesion volume (cm^3^), and **(D)** serum CA125 (Cancer Antigen 125) in the HIFU and HIFU + TCM groups. The x-axis indicates follow-up time after HIFU (baseline, 1, 3, 6, 9, and 12 months). The plotted values are unadjusted group means, and error bars represent standard errors of the mean (SEM); units are shown on each y-axis.

As shown in Figure 2A and Table 3, all patients experienced moderate-to-severe dysmenorrhoea before treatment. In the HIFU group, NRS scores decreased from 6.29 ± 1.53 at baseline to 6.22 ± 1.61, 4.69 ± 1.41, 4.18 ± 1.43, 3.49 ± 1.34 and 3.01 ± 1.12 at 1, 3, 6, 9 and 12 months after HIFU, respectively. In the HIFU + TCM group, NRS scores decreased from 6.21 ± 1.30 at baseline to 5.81 ± 1.50, 3.93 ± 1.54, 2.79 ± 1.47, 1.83 ± 0.91 and 1.64 ± 0.88 at the corresponding time points, respectively. The primary multivariable-adjusted mixed-effects model showed no significant between-group difference at 1 month (AMD = −0.41, 95% CI: −1.06 to 0.24; *P* = 0.174). However, the HIFU + TCM group had significantly lower NRS scores from 3 to 12 months (all *P* < 0.05). The direction and magnitude of the results were consistent in the IPTW-weighted sensitivity analysis.

**Table 3:** Longitudinal comparison of dysmenorrhoea NRS score, uterine volume, lesion volume, and serum CA125 between the two groups.

Time point	Variable	HIFU (*n* = 45)	HIFU + TCM (*n* = 42)	AMD	95% CI	Between-group *P* value
Pre-HIFU	NRS score	6.29 +/− 1.53	6.21 +/− 1.30	−0.08	(−0.70, 0.54)	0.718
	Uterine volume (cm^3^)	320.38 +/− 239.65	279.87 +/− 113.85	−40.51	(−118.62, 37.60)	0.607
	Lesion volume (cm^3^)	104.50 +/− 127.75	90.09 +/− 72.26	−14.41	(−55.72, 26.90)	0.970
	CA125 (U/mL)	112.59 +/− 96.51	99.98 +/− 71.51	−12.61	(−48.92, 23.70)	0.721
1 month	NRS score	6.22 +/− 1.61	5.81 +/− 1.50	−0.41	(−1.06, 0.24)	0.174
	Uterine volume (cm^3^)	284.52 +/− 179.19	242.91 +/− 112.94	−41.61	(−103.72, 20.50)	0.195
	Lesion volume (cm^3^)	88.09 +/− 99.54	71.69 +/− 58.17	−16.40	(−49.82, 17.02)	0.619
	CA125 (U/mL)	81.74 +/− 71.47	68.77 +/− 59.71	−12.97	(−38.62, 12.68)	0.316
3 months	NRS score	4.69 +/− 1.41	3.93 +/− 1.54	−0.76	(−1.35, −0.17)	0.0150
	Uterine volume (cm^3^)	225.59 +/− 154.27	181.43 +/− 83.72	−44.16	(−105.82, 17.50)	0.1650
	Lesion volume (cm^3^)	71.08 +/− 88.67	50.70 +/− 45.38	−20.38	(−52.12, 11.36)	0.2150
	CA125 (U/mL)	48.80 +/− 49.17	39.21 +/− 32.67	−9.59	(−27.82, 8.64)	0.444
6 months	NRS score	4.18 +/− 1.43	2.79 +/− 1.47	−1.39	(−1.98, −0.80)	< 0.0001
	Uterine volume (cm^3^)	179.02 +/− 112.63	131.71 +/− 66.66	−47.31	(−78.24, −16.38)	0.038
	Lesion volume (cm^3^)	51.28 +/− 61.09	33.97 +/− 35.13	−17.31	(−35.62, 1.00)	0.087
	CA125 (U/mL)	30.31 +/− 28.83	23.40 +/− 18.29	−6.91	(−17.82, 4.00)	0.535
9 months	NRS score	3.49 +/− 1.34	1.83 +/− 0.91	−1.66	(−2.15, −1.17)	< 0.0001
	Uterine volume (cm^3^)	142.17 +/− 89.88	92.72 +/− 54.30	−49.45	(−76.82, −22.08)	0.005
	Lesion volume (cm^3^)	40.02 +/− 59.46	23.34 +/− 28.34	−16.68	(−31.22, −2.14)	0.036
	CA125 (U/mL)	23.17 +/− 19.88	16.78 +/− 12.39	−6.39	(−15.82, 3.04)	0.168
12 months	NRS score	3.01 +/− 1.12	1.64 +/− 0.88	−1.37	(−1.82, −0.92)	< 0.0001
	Uterine volume (cm^3^)	119.32 +/− 89.88	68.50 +/− 42.16	−50.82	(−72.31, −29.33)	0.0005
	Lesion volume (cm^3^)	29.49 +/− 38.42	16.24 +/− 22.40	−13.25	(−22.17, −4.33)	0.010
	CA125 (U/mL)	18.48 +/− 22.48	12.42 +/− 4.17	−6.06	(−18.22, 6.10)	0.362

AMD, adjusted mean difference; CI, confidence interval. Values in table are from the primary multivariable-adjusted mixed-effects model. IPTW-weighted mixed-effects models were used only as a sensitivity analysis and produced concordant inferences.

### Changes in uterine volume and adenomyotic lesion volume after treatment

As shown in Figures 2B, C and Table 3, the primary multivariable-adjusted mixed-effects model showed no significant between-group difference in uterine volume at 1–3 months (all *P* > 0.05). From 6 to 12 months, the HIFU + TCM group showed smaller uterine volume than the HIFU group (6 months: AMD = −47.31, 95% CI: −78.24 to −16.38; *P* = 0.038; 9 months: AMD = −49.45, 95% CI: −76.82 to −22.08; *P* = 0.005; 12 months: AMD = −50.82, 95% CI: −72.31 to −29.33; *P* = 0.0005). At 12 months, the mean uterine volume reduction from baseline was 62.1% in the HIFU + TCM group and 42.7% in the HIFU group.

For lesion volume, the primary multivariable-adjusted mixed-effects model showed no significant between-group difference at 1–6 months (all *P* > 0.05). From 9 to 12 months, the HIFU + TCM group demonstrated smaller lesion volume than the HIFU group (9 months: AMD = −16.68, 95% CI: −31.22 to −2.14; *P* = 0.036; 12 months: AMD = −13.25, 95% CI: −22.17 to −4.33; *P* = 0.010), corresponding to lesion volume reduction rates of 74.9% and 71.8%, respectively. The IPTW-weighted sensitivity analysis yielded concordant inferences. These findings indicate improved lesion shrinkage during follow-up but should not be interpreted as evidence of reduced recurrence.

### Changes in cancer antigen 125 levels after treatment

As shown in Figure 2D and Table 3, serum CA125 levels progressively decreased in both groups. At 3 months, CA125 levels in both groups had decreased to within the normal range ( ≤ 47 U/mL). The primary multivariable-adjusted mixed-effects model showed no significant between-group difference at any follow-up time point (all *P* > 0.05), although the HIFU + TCM group demonstrated a slightly greater reduction at 12 months (AMD = −6.06, 95% CI: −18.22 to 6.10; *P* = 0.362). The IPTW-weighted sensitivity analysis was concordant and showed no significant between-group difference in serum CA125.

### Efficacy of traditional Chinese medicine syndrome differentiation treatment

In the HIFU + TCM group, patients with different TCM syndrome types showed varying degrees of improvement. Among patients with damp-heat and blood stasis obstruction syndrome (20 cases), the reduction in NRS score at 12 months was 4.72 ± 1.03, uterine volume decreased by 58.6% and lesion volume decreased by 51.2%. Among patients with qi stagnation syndrome (14 cases), the reduction in NRS score was 4.45 ± 0.98, uterine volume decreased by 55.3% and lesion volume decreased by 48.7%. Among patients with cold coagulation syndrome (8 cases), the reduction in NRS score was 4.31 ± 1.12, uterine volume decreased by 53.5% and lesion volume decreased by 46.9%. No significant differences in treatment efficacy were observed among the three syndrome subtypes (all *P* > 0.05; Table 4).

**Table 4:** Efficacy comparison among TCM syndrome subtypes in the HIFU + TCM group.

TCM syndrome type	*n*	12-month reduction in NRS score	Uterine volume reduction rate (%)	Lesion volume reduction rate (%)	*P* value
Damp-heat and blood stasis obstruction	20	4.72 +/− 1.03	58.6	51.2	0.821
Qi stagnation	14	4.45 +/− 0.98	55.3	48.7	0.765
Cold coagulation	8	4.31 +/− 1.12	53.5	46.9	0.693

### Recurrence

During the 12-month follow-up period, symptom recurrence occurred in 10 patients between 9 and 12 months after treatment, including 4 patients in the HIFU group (8.89%) and 6 patients in the HIFU + TCM group (14.29%). There was no significant difference in recurrence rate between the two groups (odds ratio = 1.63, 95% CI: 0.45–5.94; *P* = 0.492). Therefore, although the combined therapy was associated with improved symptom trajectories and volume reduction, it was not associated with a lower 12-month symptom recurrence rate in this cohort.

### Adverse effects

Intraoperative pain during HIFU treatment was reported in 5 patients, all of whom experienced grade 1–2 pain. No severe HIFU-related adverse events, such as skin burns, leg pain, haematuria or organ injury, were observed. In the HIFU + TCM group, mild gastrointestinal discomfort was reported in three patients (7.14%) and resolved spontaneously without interruption of treatment. No severe adverse events related to herbal medicine were reported.

## Discussion

This prospective non-randomized comparative study found that HIFU combined with syndrome differentiation-based TCM therapy was associated with more favorable 12-month trajectories of dysmenorrhoea, uterine volume, and lesion volume than HIFU alone in patients with AM. However, these associations were observed in a patient-preference cohort and should not be interpreted as proof of superior long-term efficacy or recurrence prevention.

### Innovation in traditional Chinese medicine syndrome differentiation

Many studies have demonstrated that HIFU is a safe and effective non-invasive treatment option for AM (4, 5, 20, 21, 25). However, complete ablation of adenomyotic lesions using HIFU remains challenging because of their poorly defined boundaries, resulting in residual symptoms and recurrence risk (7–9). Previous studies investigating the combination of HIFU and TCM for AM have primarily used standardized prescriptions rather than syndrome differentiation-based approaches, despite evidence suggesting that combined therapy may improve some clinical outcomes after HIFU (26–29). This reliance on uniform formulas overlooks a central principle of TCM, namely treating the same disease using different approaches according to heterogeneous syndromic presentations. In contrast, the present study classified patients into three TCM syndrome types, damp-heat and blood stasis obstruction, qi stagnation and cold coagulation, based on symptoms, tongue characteristics and pulse findings, followed by prescription of syndrome-specific herbal formulas. This approach aligns with individualized clinical care and may better address heterogeneous pathological mechanisms, although this hypothesis requires further mechanistic validation.

### Clinical efficacy of syndrome differentiation-based traditional Chinese medicine adjuvant therapy

After multivariable adjustment and IPTW sensitivity analysis, the HIFU + TCM group showed lower NRS scores at 3–12 months, smaller uterine volume at 6–12 months, and smaller lesion volume at 9–12 months compared with the HIFU group. These findings indicate that adjunctive personalized TCM may be associated with improved symptom control and lesion regression during the observed follow-up period. Notably, no significant intergroup difference was detected in 12-month symptom recurrence rates, indicating that the combined regimen did not show an advantage in preventing short-term recurrence within the current observation window.

### Synergistic mechanisms between high-intensity focused ultrasound and traditional Chinese medicine

The proposed synergistic mechanisms remain speculative because they were not directly evaluated in this study. Adenomyosis-related mechanisms involve inflammatory, neuroangiogenic, fibrotic, and molecular pathways (29–31). HIFU ablates macroscopic lesions, whereas TCM may theoretically improve microcirculation, promote absorption of necrotic tissue, regulate immune responses, and inhibit proliferation of residual ectopic cells (10, 15–18, 32, 33). The delayed emergence of between-group differences after 3 months may reflect the gradual biological effects of herbal therapy, but this interpretation requires confirmation in mechanistic studies.

### Updated literature and clinical context

Recent evidence synthesis suggests that herbal medicine as adjuvant therapy may improve short- and medium-term efficacy after HIFU for adenomyosis, mainly by relieving dysmenorrhoea and reducing lesion burden, whereas evidence for recurrence prevention remains insufficient (34). Consistent with contemporary guideline-based management of adenomyosis (3), uterus-preserving minimally invasive interventions such as HIFU are clinically important options for patients wishing to avoid hysterectomy, and recent review literature has highlighted the safety, efficacy, and fertility-preserving potential of HIFU in selected patients (35). In this context, syndrome differentiation-based herbal treatment may represent a candidate adjunctive strategy for further study rather than a practice-changing therapy at present.

### Limitations and future directions

This study has several important limitations that should be acknowledged. (1) The non-randomized patient-preference allocation design introduces potential selection bias, including unmeasured confounding factors such as treatment expectations, health beliefs, and adherence behavior, which may influence subjective outcomes such as dysmenorrhoea severity. (2) Although IPTW improved measured baseline balance, residual confounding cannot be excluded. (3) This was an exploratory study providing preliminary associative evidence rather than definitive proof of efficacy or causality. (4) The relatively small sample size limited the precision of subgroup analyses and may increase the risk of model instability despite the use of a parsimonious propensity model and stabilized trimmed weights. (5) The 12-month follow-up period may be insufficient to evaluate long-term recurrence in a chronic condition such as AM. (6) The use of individualized herbal adjustments reduced intervention standardization and may limit reproducibility. (7) The study did not assess several clinically relevant outcomes, including menorrhagia, anemia, quality of life, fertility, and reproductive outcomes. (8) No molecular or inflammatory biomarkers were evaluated, and therefore the proposed biological mechanisms remain hypothetical.

Future research should (1) conduct adequately powered randomized controlled trials to confirm whether the observed associations reflect causal treatment effects; (2) extend follow-up duration to evaluate durability and recurrence beyond 12 months; (3) incorporate prespecified balance diagnostics and transparent weighting reports in comparative observational analyses; (4) standardize core TCM treatment frameworks while preserving syndrome differentiation; and (5) include broader patient-reported and reproductive outcomes.

## Conclusions

In summary, this prospective non-randomized comparative study showed that HIFU combined with syndrome differentiation-based individualized TCM herbal therapy was associated with greater improvement in dysmenorrhoea and greater reductions in uterine and lesion volumes during 12-month follow-up compared with HIFU alone. No significant difference was observed in 12-month symptom recurrence between groups. These findings support further investigation of personalized TCM as an adjunct to HIFU but should not be interpreted as evidence of reduced recurrence or as sufficient to inform clinical guideline changes without confirmation in large-scale randomized trials.

## Data Availability

The original contributions presented in the study are included in the article/Supplementary material, further inquiries can be directed to the corresponding author.

## Supplementary material

Supplementary Figure 1 Representative ultrasound and MRI (magnetic resonance imaging) images of adenomyotic lesions at baseline and 12 months.
